# Color Management in Digital Pathology

**DOI:** 10.1155/2014/652757

**Published:** 2014-12-30

**Authors:** W. Craig Revie, Mike Shires, Pete Jackson, David Brettle, Ravinder Cochrane, Darren Treanor

**Affiliations:** ^1^FFEI Ltd., Hemel Hempstead HP2 7DF, UK; ^2^University of Leeds, Leeds LS2 9JT, UK; ^3^Leeds Teaching Hospitals NHS Trust, Leeds LS14 6UH, UK

## Abstract

In digital microscopes and whole slide imaging systems, images of slides are captured, transmitted, and reproduced on a computer display. In order to allow pathologists to interpret these images accurately and efficiently, it is important that colors from the slides are displayed in a consistent and reliable fashion.

The final color of the image presented to the viewing pathologist depends on several steps through the imaging pathway, including sample illumination, magnification, image capture, compression, storage, and reproduction on the computer display. There are many possible system designs and, within a single system, different setup options which can affect the final image leading to significant variation in image appearances.

This paper summarizes recent work by members of the International Color Consortium Medical Imaging Working Group to develop test materials and methods for the assessment of color calibration of digital microscope systems. This work includes sharing of ideas on device calibration and image processing and display.

The paper further discusses the challenges encountered in the development of a suitable color target that includes a set of patches with spectra similar to those encountered when viewing pathology slides with stained tissue samples.

## Background

Staining of tissue sections on glass slides is the foundation on which diagnosis and prognosis in pathology are based.

In digital microscopes and whole slide imaging (WSI) systems, images of slides are captured, transmitted, and reproduced on a computer display. In order to allow pathologists to interpret these images accurately and efficiently, it is important that colors from the slides are displayed in a consistent and reliable fashion.

**Figure 1:**
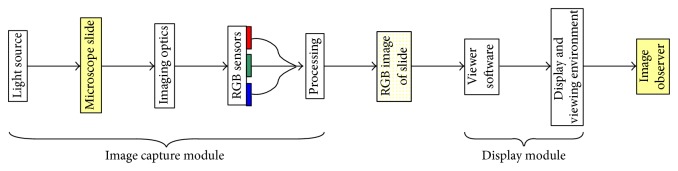
WSI image processing.

The final color of the image presented to the viewing pathologist depends on several steps through the imaging pathway, including sample illumination, magnification, image capture, compression, storage, and reproduction on the computer display as shown in Figure 1. There are many possible system designs and, within a single system, different setup options which can affect the final image leading to significant variation in image appearances.

We have proposed an objective measure for the color performance of digital microscope systems which allows the overall result of this processing sequence to be assessed.

## Color Calibration Assessment for Pathology

Recognizing the importance of color reproduction, several digital pathology vendors and research groups have attempted to develop color calibration systems for pathology images.

Silverstein et al. developed ICC color calibration profiles for three displays focusing on the display end of the imaging workflow [1] using an on-screen color target. The resulting profiles can be used to compare display performance, but as they do not include measurement of the color variation introduced by the imaging device (WSI instrument or digital microscope), they do not allow for end-to-end color calibration.

Others have used color calibration targets based on synthetic targets such as photographic transparency of the Macbeth color target. Such film based methods involve imaging the target with the imaging device and then using the resulting image to develop a color correction profile specific to that device [2, 3]. Although these methods almost certainly produce improvements in color consistency between scanners, they are limited by the fact that the colors are produced by combinations of cyan, magenta, and yellow film dyes which have substantially different spectra from those of histopathology stains. This difference is very likely to lead to substantial errors.

To address these limitations, tissue based color targets have been proposed such as a section of mouse embryo stained in a standard way. Although such targets have the advantage of accurately representing the target material to be imaged, producing useful color phantoms based on stained tissue is difficult due to the significant variation between stained slides.

A fundamental problem when assessing the way in which a digital microscope system handles color is that of metamerism. This is a well-known effect present in many situations where two colors appear identical in one situation but appear to be different in another.

## Calibration Assessment Target

**Figure 2:**
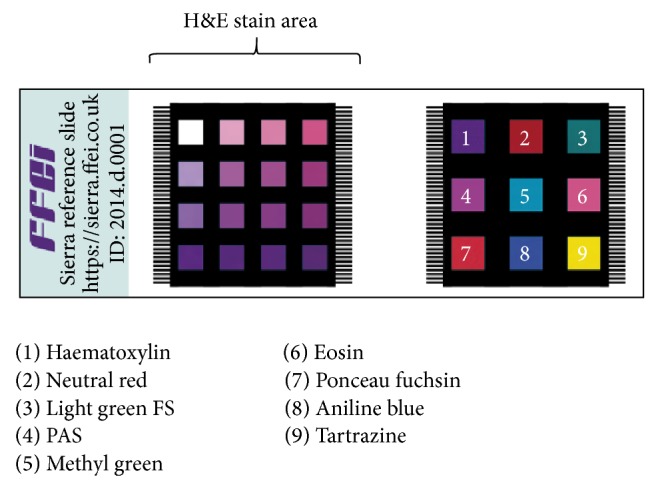
WSI image processing.

We have developed a novel color calibration assessment target for digital pathology as shown in Figure 2 which allows the color calibration of digital microscope systems to be measured objectively. The target includes a representative set of colored patches of biopolymer that have been stained with the pathology stains.

## Limitations and Future Work

The choice of stains used in this work reflects the UK's histopathology and cytopathology practice. This is assumed to be broadly representative of worldwide pathology practice. There may be significant international variability in both the stains used and the exact chemical composition of the dyes used—for example, we analyzed both Meyer's and Harris's haematoxylin, but other methods may be in use which have not been measured in this work. Although the pilot work described above produced a color calibration assessment target with suitable characteristics for use, currently undergoing testing, further development will likely be needed to manufacture this device on a large scale.
